# Opposing Effects of Omega-3 and Omega-6 Long Chain Polyunsaturated Fatty Acids on the Expression of Lipogenic Genes in Omental and Retroperitoneal Adipose Depots in the Rat

**DOI:** 10.1155/2010/927836

**Published:** 2010-08-05

**Authors:** B. S. Muhlhausler, R. Cook-Johnson, M. James, D. Miljkovic, E. Duthoit, R. Gibson

**Affiliations:** ^1^FOODplus Research Centre, School of Agriculture, Food and Wine, The University of Adelaide, Adelaide 5064, Australia; ^2^Division of Health Sciences, Sansom Institute for Health Research, University of South Australia, Adelaide SA 5001, Australia; ^3^Rheumatology Unit, School of Medicine, The University of Adelaide, Adelaide SA 5005, Australia

## Abstract

This study aimed to determine the effect of varying dietary intake of the major n-3 PUFA in human diets, *α*-linolenic acid (ALA; 18 : 3n-3), on expression of lipogenic genes in adipose tissue. Rats were fed diets containing from 0.095%en to 6.3%en ALA and a constant n-6 PUFA level for 3 weeks. Samples from distinct adipose depots (omental and retroperitoneal) were collected and mRNA expression of the pro-lipogenic transcription factors Sterol-Retinoid-Element-Binding-Protein1c (SREBP1c) and Peroxisome Proliferator Activated Receptor-*γ* (PPAR*γ*), lipogenic enzymes Sterol-coenzyme Desaturase1 (SCD-1), Fatty Acid Synthase (FAS), lipoprotein lipase (LPL) and glycerol-3-phosphate dehydrogenase (G3PDH) and adipokines leptin and adiponectin determined by qRT-PCR. Increasing dietary ALA content resulted in altered expression of SREBP1c, FAS and G3PDH mRNA in both adipose depots. SREBP1c mRNA expression was related directly to n-6 PUFA concentrations (omental, *r* ^2^ = .71; *P* < .001; Retroperitoneal, *r* ^2^ = .20; *P* < .002), and inversely to n-3 PUFA concentrations (omental, *r* ^2^ = .59; *P* < .001; Retroperitoneal, *r* ^2^ = .19; *P* < .005) independent of diet. The relationship between total n-6 PUFA and SREBP1c mRNA expression persisted when the effects of n-3 PUFA were controlled for. Altering red blood cell concentrations of n-3 PUFA is thus associated with altered expression of lipogenic genes in a depot-specific manner and this effect is modulated by prevailing n-6 PUFA concentrations.

## 1. Introduction

In adults, changes in the patterns of expression of key regulatory and functional genes within adipose tissue are important determinants of fat accumulation and can profoundly influence the ability of an individual to maintain energy balance and resist weight gain [1–4]. The transcription factors Sterol Retinoid Binding Protein-1c (SREBP1c) and Peroxisome Proliferator Activated Receptor-*γ* (PPAR*γ*) regulate lipid storage and adipose tissue mass by regulating the expression of genes in the lipogenic pathway. Specifically, activation of SREBP1c and PPAR*γ* increases the expression of a series of enzymes which increase the synthesis and storage of triglycerides in adipose tissue, including lipoprotein lipase (LPL), which increases uptake of fatty acids from the circulation, and Fatty Acid Synthase (FAS) and glycerol-3-phosphate dehydrogenase (G3PDH), which both promote triglyceride synthesis [5]. 

While the cause of the current global obesity epidemic includes excessive food consumption and reduced exercise, there is increasing evidence that both the quantity and the type of fats in the diet have a major role in defining the propensity of an individual to accumulate excess body fat. It is acknowledged that diets high in saturated fat (>45%) promote fat accumulation [6], and low-fat diets are commonly recommended as a means of weight-loss [7]. It is also clear, however, that polyunsaturated fatty acids (PUFAs), particularly those of the omega-3 (n-3) and omega-6 (n-6) classes, also have the ability to influence the rate of lipid accumulation. There is evidence which suggests that n-6 PUFAs promote, while n-3 PUFAs inhibit, lipid accumulation within adipose depots, and this has led to the suggestion that the increasing dominance of n-6 over n-3 PUFA in typical Western diets [8] may be one factor contributing to the increasing incidence of obesity [5, 9]. Conversely, it has been postulated that increased dietary intake of n-3 and/or decreased intake of n-6 fatty acids may have benefits in relation to suppressing fat deposition. There is also evidence that supplementing the diet of rodents [10] and humans [11] with n-3 LCPUFA may promote loss of body fat. However, there are only a handful of studies which have investigated in any detail the impact of altering the balance of n-3 and n-6 PUFAs in the diet on the expression of lipogenic genes in adipose tissue, and which have determined the specific n-3 and n-6 PUFAs which have the greatest contribution to these effects.

The majority of A Western diets are low in omega-3 LCPUFA as a consequence of a low fish intake, and the major source of n-3 fats in these diets is from vegetable oils in the form of *α*-linolenic acid (ALA; 18 : 3n-3). In this study, we therefore examined the effect of a wide range of dietary intakes of ALA with the level of the main n-6 fatty acid found in vegetable oils, linoleic acid (LA; 18 :  2n-6), kept constant. The primary outcome measure was the mRNA expression of key lipogenic genes within two of the principal fat depots in the rodent, the abdominal (omental) and retroperitoneal depots. An important secondary aim was to determine whether the circulating concentrations of any specific fatty acid could predict the level of mRNA expression SREBP1c mRNA within each of these adipose depots.

## 2. Methods

### 2.1. Animals and Diet

All procedures were approved by the Animals Ethics committee of the University of Adelaide. All diets consisted of a base feed (SF00-229, Specialty Feeds, Glen Forrest, WA) which was blended with different mixtures of flaxseed, macadamia, sunflower oils in order to generate diets in which ALA made up between 0.095% and 6.3% of total energy (%en). The total percentage of fat was held constant at 5% v/v in all diets. All feeds were tested to ensure consistency of the fatty acid composition prior to starting the experiment. The base diet and the proportion of LA in the diet was kept constant at 2.1%en in all diets, so that only the ALA content was varied. Detailed information on the composition of the experimental diets is shown in Table 1. 

Forty (40) 6-week old Female Dark Agouti rats were initially fed for a 3 week feed-in period on a diet which contained 100% Macadamia oil in order to ensure that all animals had a comparable baseline fatty acid status at the time of commencing the dietary treatment. This feed-in diet contained 0.016%en ALA and 0.27%en LA. Following the feed-in period, rats were randomly assigned to one of the 8 experimental diets (5 animals per dietary treatment) and were maintained on these diets for 3 weeks. Food was replenished daily and water was available *ad libitum*. The animals were group housed in an animal facility maintained on a 12 hr light/12 hour dark cycle at a temperature of 20–22°C. There was no difference in body weight of rats in the different experimental groups either at the start of the dietary intervention, or following the 3-weeks of feeding. 

At the end of the 3 week period, all rats were humanely killed by an overdose of anesthetic (Fluothane, ICI, Melbourne, Vic, Australia). A blood sample was collected by cardiac puncture for the subsequent measurement of plasma, and erythrocyte fatty acid composition. A sample of adipose tissue from the omental adipose depot and the retroperitoneal depot was collected from each animal and stored in RNAlater at −20°C for subsequent measurement of gene expression. The sample was collected by the same investigator on each occasion in order to ensure consistency of the sampling site.

### 2.2. Lipid Extraction

All solvents used in this study were analytical grade and were purchased from Ajax Finechem Pty Ltd (Auckland, New Zealand) or Chem-Supply (South Australia, Australia). Other chemicals and reagents were purchased from Sigma-Aldrich (St. Louis, MO) unless specified otherwise. The total lipids of the experimental oil formulation, plasma and erythrocytes were extracted following the protocol of Bligh and Dyer [12] using chloroform-methanol (2 : 1, v/v). The phospholipids were separated from total lipid extracts by thin layer chromatography (TLC) on silica gel plates (Silica gel 60H; Merck, Darmstadt, Germany). A lipid classes standard 18-5 (NU-CHEK Prep; Elysian, MN) was run on the plates for lipid identification. The mobile phase for TLC was petroleum spirit/acetone (3 : 1, v/v). The TLC plates were sprayed with fluorescein 5-isothiocyanate in methanol, and the lipid classes present were then visualized under UV light. The phospholipid bands located at the bottom of TLC were transferred into a vial containing 1% sulphuric acid (H_2_SO_4_) in methanol for transmethylation. All solvents used for extraction and separation contained 0.005% (w/v) antioxidant, butylated hydroxyl toluene (BHT).

### 2.3. Fatty Acid Methylation

All lipids and phospholipids were transesterified with 1% H_2_SO_4_ in methanol at 70°C for 3 h. After the samples were cooled, the resulting fatty acid methyl esters (FAME) were extracted with *n*-heptane and transferred into vials containing a scoop of anhydrous sodium sulphate (Na2SO_4_).

### 2.4. Gas Chromatographic Analysis of FAME

FAME were separated and quantified by GC (Hewlett-Packard 6890; Palo Alto, CA) equipped with a capillary column (50 m × 0.32 mm id) coated with 0.25 *μ*m film thickness silica (BPX-70; SGC Pty Ltd, Victoria, Australia) and a flame ionisation detector (FID). The injector temperature was set at 250°C and the FID temperature at 300°C. The oven temperature at injection was initially set at 140°C and was programmed to increase to 220°C at a rate of 5°C per minute. Helium gas was utilized as a carrier at a flow rate of 35 cm per second in the column. The identification and quantification of FAMEs were achieved by comparing the retention times and peak area % values of unknown samples to those of commercial lipid standards (Nuchek Prep Inc, Elysian, USA) using the Hewlett-Packard Chemstation data system. FAMES standards for unique n-3 LCPUFAs 24 : 5 n-3 and 24 : 6 n-3 were purchased from Larodan Fine Chemicals (Malmö, Sweden).

### 2.5. Isolation of RNA, Production of cDNA, and qRT-PCR Analysis

RNA was extracted from 100 mg adipose tissue from each depot (Trizol reagent, Invitrogen Australia Pty Limited, Australia) from all experimental animals. RNA was purified using the RNeasy Mini Kit (QIAGEN, Basel, Switzerland). The quality and concentration of the RNA was determined by measuring the absorbance at 260 and 280 nm, and RNA integrity was confirmed by agarose gel electrophoresis. cDNA was then synthesised using the purified RNA (~2 *μ*g) and Superscript III reverse transcriptase (Invitrogen Australia Pty Limited, Mount Waverley, Australia) with random hexamers.

The relative expression of mRNA transcripts of the SCD-1, PPAR*γ*, SREBP1c, FAS, LPL, G3PDH, leptin, and adiponectin mRNA transcripts (Table 2), and the housekeeper gene *β*-actin (Rn-Actb-1-SG, QuantiTect Primer Assay, Qiagen) were measured by quantitative real time reverse transcription-PCR (qRT-PCR) using the Sybr Green system in an ABI Prism 7500 Sequence Detection System (PE Applied Biosystems, Foster City, CA). Each amplicon was sequenced to ensure the authenticity of the DNA product and qRT-PCR melt curve analysis was performed to demonstrate amplicon homogeneity. Each qRT-PCR reaction well (10 *μ*L total volume) contained: 6 *μ*L 2x Sybr Green Master Mix (PE Applied Biosystems, Foster City, CA): 1 *μ*L of each primer giving a final concentration of 450 or 900 nM, 2.0 *μ*L of molecular grade H_2_O, and 1.0 *μ*L of a 50 ng/*μ*L dilution of the stock template. Controls containing no reverse transcriptase were also used.

The abundance of each mRNA transcript was measured and expression relative to that of *β*-actin was calculated using the comparative threshold cycle (*C*_*t*_) method (Q-gene qRT-PCR analysis software).

### 2.6. Statistics

The effect of dietary treatment on erythrocyte and plasma phospholipid fatty acid composition were determined by one-way ANOVA. The Duncan's multiple range test was used post hoc to identify differences between mean values. The effect of diet on gene expression in omental and retroperitoneal adipose tissue was similarly determined. Stepwise multiple linear regression was used to determine the strongest predictor of adipocyte gene expression in the omental and retroperitoneal adipose depots. Relationships between variables were determined using linear regression analyses. Where there were potential associations between independent variables, multiple linear regression analyses were used and partial correlation coefficients were derived. All data are presented as the mean ± SEM and a probability of <5% (*P* < .05) was taken as significant.

## 3. Results

### 3.1. Animals

#### 3.1.1. Composition of Red Blood Cell (RBC) and Plasma Phospholipids

The ALA content of RBC phospholipids as a % of total lipids increased progressively with increasing dietary ALA (Table 3). Dietary ALA was associated with increased ALA elongation and desaturation products, Eicosapentaenoic acid (EPA; 20 : 5n-3), Docosapentaenoic acid (DPA; 22 : 5n-3), and Docosahexaenoic acid (DHA; 22 : 6n-3). Whereas EPA and DPA increased proportionally with increasing dietary ALA, DHA displayed a different relationship (Table 3). The DHA content of RBC phospholipids increased modestly to a peak at a dietary ALA content of 0.76%en, but then decreased with further increases in dietary ALA content (Table 3). 

The RBC phospholipid content of arachidonic acid (AA, 20 : 4n-6), the derivative of LA, decreased in a linear manner with increasing dietary ALA content (Table 3).

Overall, the total n-3 PUFA content of the RBC phospholipids increased, and total n-6 PUFA content decreased with increasing dietary ALA content (Table 3). 

The plasma phospholipid fatty acid changes were similar to those of the RBC phospholipids (data not shown).

#### 3.1.2. Expression of Adipocyte Genes

**Omental Depot**

Dietary ALA altered the omental fat content of mRNA for the transcription factor SREBP1 (*F* = 5.36, *P* < .0001), and the lipogenic enzymes SCD1 (*F* = 5.22, *P* < .0001), FAS (*F* = 7.66, *P* < .0001), and G3PDH (*F* = 12.87, *P* < .0001). The expression of these genes increased with increasing dietary ALA, reaching maximal levels at 0.38%en to 0.76%en ALA. However, the mRNA expression of all these genes decreased with further increases in dietary ALA (Table 4). The effect of diet on leptin mRNA expression in omental fat showed a similar pattern, but the changes in mRNA expression did not reach statistical significance (Table 4). There was no significant effect of diet on mRNA expression of PPAR*γ*, LPL, or adiponectin in the omental fat depot (Table 4).

The expression of FAS, G3PDH, and leptin mRNA were each directly related to the expression of SREBP1c in omental adipose tissue (FAS mRNA = 116 SREBP1c mRNA + 0.01, *r* ^2^ = 0.28, *P* < .001; G3PDH mRNA = 132 SREBP1c mRNA + 0.01, *r* ^2^ = 0.25, *P* < .001; Leptin mRNA = 21 SREBP1c mRNA + 0.003, *r* ^2^ = 0.38, *P* < .001). The expression of adiponectin, LPL, FAS and leptin mRNA was also directly related to PPAR*γ* mRNA expression in the omental adipose depot (adiponectin mRNA = 51.1 PPAR*γ* mRNA + 0.24, *r* ^2^ = 0.39, *P* < .001; LPL mRNA = 32.3 PPAR*γ* mRNA + 0.72, *r* ^2^ = 0.23, *P* < .02; FAS mRNA = 3.5 PPAR*γ* mRNA + 0.01, *r* ^2^ = 0.163, *P* < .05). The expression of G3PDH mRNA also tended (*P* = .06) to be directly related to the expression of PPAR*γ* mRNA.

**Retroperitoneal Depot**

There was no difference in the expression of SCD-1 or leptin mRNA between dietary treatments (Table 4). The expression of SREBP1c mRNA in retroperitoneal fat decreased (*F* = 2.27, *P* < .05) with increasing dietary ALA content (Table 4). FAS mRNA expression was highest when dietary ALA content was less than 2%en, after which it decreased with increasing dietary ALA content (*F* = 9.11, *P* < .001; Table 4). G3PDH mRNA expression followed a similar pattern to that of FAS mRNA (*F* = 3.88, *P* < .01; Table 4).

#### 3.1.3. Determinants of Adipocyte Gene Expression

**Omental Depot**

**Figure 1:**
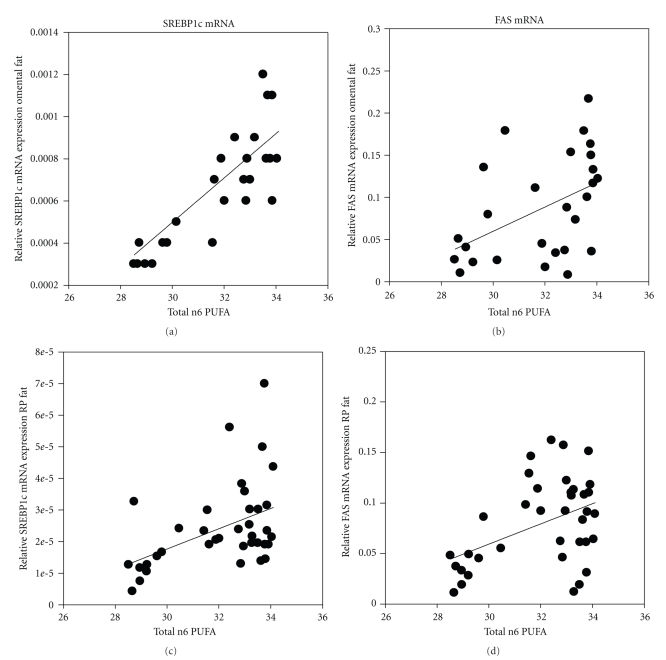
The relationship between total n-6 concentration in erythrocyte phosphosolipids and the expression of (a) and (c) SREBP1c mRNA and (b) and (d) FAS mRNA in omental (a) and (b) and retroperitoneal (c) and (d) adipose tissue. There was a significant positive relationship between SREBP1c mRNA expression and total n-6 concentration in both omental and retroperitoneal fat. There was also a weak but significant positive relationship between FAS mRNA expression and total n-6 PUFA content in both omental and retroperitoneal adipose tissue.

The expression of SREBP1 mRNA in omental fat was related to n-3 LCPUFA status as measured in RBC. There was an inverse relationship between RBC EPA (*r* ^2^ = 0.62, *P* < .0001), DPA (*r* ^2^ = 0.59, *P* < .0001), total n-3 LCPUFA (*r* ^2^ = 0.59, *P* < .0001), and SREBP1 mRNA. SREBP1c mRNA expression was unrelated to DHA content of RBC phospholipids. The expression of SREBP1c mRNA was positively related to AA content (*r* ^2^ = 0.66, *P* < .0001) and total n-6 content (*r* ^2^ = 0.71, *P* < .0001) of RBC phospholipids. In stepwise multiple linear regression analysis, the total n-6 PUFA content of RBC phospholipids emerged as the strongest independent predictor of SREBP1c gene expression in omental fat (Figure 1(a)). The relationship between total RBC phospholipids n-6 content and SREBP1c gene expression was positive and persisted when the effects of n-3 PUFAs were controlled for in the analysis.

The expression of FAS and LPL mRNA was also inversely related to n-3 LCPUFA (FAS, *r* ^2^ = 0.16, *P* < .01; LPL, *r* ^2^ = 0.14, *P* < .05) and positively related to total n-6 PUFA content (FAS, *r* ^2^ = 0.24, *P* < .001; Figure 1(b); LPL, *r* ^2^ = 0.16, *P* < .05). There was no significant relationship between either n-3 or n-6 PUFA or LCPUFA content and the mRNA expression of PPAR*γ*, G3PDH, leptin or adiponectin mRNA in omental fat. The strongest predictor of FAS, G3PDH, and leptin expression in omental fat was SREBP1c mRNA expression, and any relationships between the mRNA expression of these genes and fatty acid content of RBC or plasma phospholipids did not persist when adjustment was made for SREBP1c mRNA expression.

**Retroperitoneal Depot**

In the retroperitoneal depot, SREBP1c mRNA expression was inversely related to ALA (*r* ^2^ = 0.20, *P* < .005), EPA (*r* ^2^ = 0.22, *P* < .005) and DPA (*r* ^2^ = 0.19, *P* < .005) content of RBC phospholipids, and to total n-3 LCPUFA content (*r* ^2^ = 0.19, *P* < .005). SREBP1c mRNA expression was positively related to AA (*r* ^2^ = 0.21, *P* < .005) and total n-6 content (*r* ^2^ = 0.20, *P* < .005, Figure 1(c)). Multiple linear regression analysis showed that the EPA content of RBC phospholipids was the strongest independent predictor of SREBP1c mRNA expression in retroperitoneal adipose tissue, however this relationship did not persist when the effects of n-6 PUFA were controlled for in the analysis. Interestingly, we found that there was no inverse relationship between SREBP1c mRNA expression and the DHA content of RBC phospholipids, and in fact there was a weak but statistically significant positive relationship between DHA content and SREBP1c mRNA expression in retroperitoneal fat (*r* ^2^ = 0.11, *P* < .005).

A summary of all relationships between SREBP1c mRNA expression and fatty acid content in erythrocyte phospholipids is presented in Table 5.

There was a weak positive relationship between total n-6 PUFA content and FAS mRNA expression in the retroperitoneal adipose depot (*r* ^2^ = 0.12, *P* < .01, Figure 1(c)). As in the omental adipose depot, the strongest predictor of FAS, G3PDH and leptin mRNA expression in retroperitoneal fat was SREBP1c mRNA expression, and any relationships between the mRNA expression of these genes and fatty acid content of RBC or plasma phospholipids did not persist when adjustment was made for SREBP1c mRNA expression.

## 4. Discussion

Our finding that the mRNA expression of the key lipogenic transcription factor, SREBP1c, was inversely related to the concentration of the three potent n-3 PUFA, ALA, EPA and DPA and positively related to n-6 PUFA concentration extend previous findings that, in addition to their effects on fat oxidation in adipose tissue, n-3 and n-6 fatty acids also modulate the expression of lipogenic genes within adipose depots [5, 13, 14]. Importantly, we demonstrated that fat depots in different anatomical locations have different degrees of responsiveness to altered levels of n-3 and n-6 PUFA LCPUFA in the body, with effects being more pronounced in the omental adipose tissue compared to the visceral retroperitoneal depot. This has clear clinical implications, since the excess accumulation of omental adipose tissue in both humans and animal models is recognized as the major risk factor for the development of metabolic disease [15]. Whilst previous studies have shown different effects between distinct visceral and subcutaneous adipose depots in the response to increasing n-3 PUFA admixed to high fat diets [10], our current study is the first to demonstrate a differential response between fat depots to n-3 and n-6 PUFA in the absence of a background of high-fat feeding. 

Although there were strong relationships between SREBP1c mRNA expression and both the total n-3 and total n-6 concentrations, we found that the strongest predictor of SREBP1c mRNA in omental adipose tissue was the total content of n-6 PUFA in the phospholipids of the red blood cells, and the inverse relationship between n-3 content and SREBP1c expression did not persist when adjustment was made for total n-6 levels. The results of the present study therefore confirm previous findings which have demonstrated a prolipogenic role for n-6 PUFA, in particular AA, both *in vitro* and *in vivo* [14, 16]. AA potently stimulates adipocyte differentiation/proliferation through activation of the proadipogenic transcription factor, PPAR*γ* [14], and feeding a high LA diet during the suckling period is associated with an increased accumulation of epididymal adipose tissue in rodents at 8 weeks of age [13]. The results of previous studies have led researchers to speculate that n-3 LCPUFA act to suppress the adipogenic and lipogenic effects of n-6 PUFA. Whilst the balance of n-3 and n-6 PUFA in the diet are likely to be important in defining its lipogenic potential, the results of the present study suggest that the n-6 PUFA content of erythrocyte phospholipids, and therefore the ability of diets to alter this, may be the most important factor which defines a diet's lipogenic potential. An alternate possibility may be that the increase in dietary intake of ALA acted both to reduce n-6 PUFA content in red blood cell and plasma phospholipids and suppress lipogenic activity in adipose tissue through separate pathways, and that there is in fact no causal relationship between these two factors. This is unlikely, however, given the existing evidence from *in vitro* and *in vivo* animal studies which support the proadipogenic action of the n-6 PUFA. The findings of this study also suggest that the anti-lipogenic effects of n-3 PUFAs are less potent than the prolipogenic actions of n-6 fatty acids across the range of dietary fatty acids concentrations in the present study. 

This finding has important implications, since typical diets in much of the Western world are currently characterized by high levels of n-6 PUFA, and n-6 PUFA are present at high levels in many common cooking oils and processed foods [8]. These findings are supported by the series of elegant studies by Lands and colleagues, in which they showed that the fatty acid composition in phospholipids of human and rodent plasma could be related to the dietary intake of LA (n-6) and ALA (n-3) by empirical hyperbolic equations, such that higher intake of LA was associated with reduced conversion of ALA to downstream long-chain derivatives [17, 18]. It would therefore appear that any attempt at limiting fat deposition by supplementing the diet with n-3 LCPUFA will need to be accompanied by a reduction in n-6 PUFA consumption in order to be effective.

We also found that there were relationships between n-3 and n-6 PUFA concentrations and the mRNA expression of the lipogenic enzyme FAS in both omental and retroperitoneal adipose tissue. These correlations were, however, substantially weaker than the relationships with SREBP1c mRNA expression in the respective depot, and were explained entirely by the influence of SREBP1c on FAS mRNA expression. SREBP1c mRNA was directly related to the expression of all lipogenic genes we investigated in both the omental and retroperitoneal depots. This is in line with the known role of SREBP1c as the master regulator of lipogenesis, driving the expression of a number of enzymes which play a central role in lipogenesis, including those investigated in the current study [19]. These results therefore suggest that alterations in SREBP1c mRNA in response to altered dietary fatty acid content is likely to mediate the effect of dietary fatty acids on lipogenic activity of adipose depots.

Due to the relatively strong relationships between erythrocyte fatty acid content and SREBP-1c mRNA expression in the omental fat depot, we also determined the impact of the different n-3 and n-6 dietary content on the expression of PPAR*γ*, another key lipogenic transcription factor in adipose tissue. Interestingly, we found no difference in the expression of PPAR*γ* mRNA in the omental adipose tissue between different dietary treatments, and PPAR*γ* mRNA was also not related to erythrocyte content of either n-6 or n-3 fatty acids. These data may suggest that the principal pathway through which fatty acids act to modulate the expression of lipogenic genes in through altered expression of SREBP-1c, and consequent activation of the downstream cascade, rather than through effects on PPAR*γ*. This is in line with previous findings, which suggest that the n-3 fatty acids act to increase PPAR*γ* activity in target tissues, and that this is associated with increased insulin sensitivity [5]. An alternate explanation is that the effects of fatty acids on PPAR*γ* signaling are mediated through changes in PPAR*γ* acitvtiy, rather than changes in gene expression, which was not measured in this study, since fatty acids have been shown to act as natural ligands of the PPAR*γ* gene in other studies [20]. Nevertheless, the positive relationships between PPAR*γ* mRNA and the expression of the lipogenic enzymes, LPL, FAS, and G3PDH in this study provides evidence that PPAR*γ* contributes to the regulation of adipocyte gene expression in this study. We also found a strong relationship between the expression of PPAR*γ* and mRNA expression of the insulin-sensitivity adipokine, adiponectin [21], in the omental adipose depot, which suggests that PPAR*γ* may play a more important role in regulation of insulin sensitivity, compared to lipogenesis. 

In previous studies, the fat reducing or anti-lipogeneic effects of n-3 PUFAs have generally been attributed to the actions of both of the potent long-chain n-3 PUFAs, EPA and DHA [5, 11]. In the present study, both EPA and DPA concentrations were increased whilst there was no appreciable change in DHA concentrations, and we were therefore able to separate the effects of DHA from those of the other n-3 LCPUFA, DPA and EPA. These effects are consistent with those which have previously been reported in adult humans, in which feeding healthy subject a diet high in ALA was associated with an increase in the EPA in tissues to concentrations comparable with those associated with fish-oil supplementation, with no appreciable change in tissue DHA concentrations [22]. The similarity in effect of increased dietary ALA content on tissue fatty acid concentrations in rodents and humans supports the clinical relevance of our rodent model. 

We were thus able to demonstrate that total n-3 concentrations are inversely associated with alterations in adipocyte gene expression in the absence of increases in DHA concentrations. Furthermore, we found that SREBP1c mRNA expression in both omental and retroperitoneal fat was inversely related to tissue DPA and EPA concentrations. Collectively, these findings suggest that DPA and EPA, as well as DHA, contribute to the n-3 PUFA-mediated suppression of lipogenic activity in adipose tissue *in vivo*. The relative potency of DPA, DHA and EPA in suppressing lipogenic gene expression and curtailing fat deposition is clearly an important question which remains to be elucidated, and calls for studies in which the concentrations of DPA, DHA and EPA are altered independently. 

The results of the red blood cell analyses for this study are consistent with previous data showing that there is a nonlinear relationship between DHA synthesis and dietary ALA content. We found that whilst increasing dietary ALA content from 0.095 to 1%en was associated with a modest increase in DHA content of red blood cell phospholipids, further increasing the dietary ALA content actually resulted in a decrease in DHA. This is similar to the results of previous studies in piglets [23] and in rat pups, which also reported a curvilinear relationship between dietary ALA and tissue DHA content. In contrast to DHA, DPA and EPA content in red blood cell phospholipids increased in a linear fashion as dietary ALA content increased. Furthermore, both DPA and EPA content was directly related to the total n-3 and n-3 LCPUFA content of the red blood cell phospholipids, whilst DHA content was not related to either of these measures. Collectively, these findings suggest that the limitation to the synthesis of DHA occurs at a step in the biosynthetic pathway beyond the production of DPA, that is, the final elongation or desaturation step in this pathway. It would therefore appear that one or both of these enzymes becomes saturated at higher levels of dietary ALA and thereby limit further synthesis of DHA. This is likely to be a consequence of the higher levels of ALA and other intermediate length n-3 PUFA, since each of these enzymes has several competing substrates, and previous studies have shown that increased competition from these substrates limits their availability for DHA synthesis. 

In summary, we have shown that modulating dietary ALA content can result in altered patterns of gene expression within adipose cells in distinct anatomical locations. We have also shown that the level of gene expression, independent of dietary treatment, is related positively to n-6 and inversely to n-3 concentrations, and that the proadipogenic effects of n-6 PUFA may be more potent than the anti-lipogenic effects of n-3 fatty acids in omental adipose tissue. These findings suggest that reducing the n-6 content of the diet, in addition to increasing n-3 intake, may be required in order to optimise the ability of specific diets to suppress fat accumulation and aid loss of body fat. These data highlight the need for further studies which examine in more detail the relative impact of increasing dietary n-3 content and reducing dietary n-6 content on lipogenic gene targets in adipose tissue *in vivo*, in order to identify optimal PUFA targets for limiting or reducing fat deposition in humans.

##  Authors' Contribution 

M. James, R. Cook-Johnson and R. Gibson were responsible for the design of the research project. R. Cook-Johnson, B. S. Muhlhausler and E. Duthoit undertook the hands-on conduct of the experiments and data collection. B. S. Muhlhausler and R. Gibson analyzed data and wrote the first draft of the paper. All authors read and approved the final paper.

## Figures and Tables

**Table 1:** Dietary fatty acid composition of the experimental diets.

	6.3%en ALA	4.1%en ALA	2.05%en ALA	1.00%en ALA	0.76%en ALA	0.38%en ALA	0.19%en ALA	0.095%en ALA
18 : 3n-3 (ALA)	47.9	31.6	15.7	7.5	5.8	3.0	1.6	0.9
18 : 2n-6 (LA)	16.6	17.5	17.4	16.7	17.5	17.5	17.0	17.0
Total SFA	12.2	14.0	15.8	16.3	17.3	17.6	18.0	17.7
Total MUFA	0.2	0.2	0.3	0.3	0.4	0.4	0.6	0.7
Total n-9 PUFA	0.3	1.0	1.7	1.9	2.1	2.2	2.3	2.4

All values are expressed as a % of total lipids by volume; all values are obtained from GC analysis of the feed content after manufacture.

**Table 2:** Primers sequences used for the determination of gene expression in adipose tissue by qRT-PCR.

GENE	Forward Primer (5′–3′)	Reverse Primer (5*'*–3*'*)	Accession No.
SREBP1c	TGCGGACGCAGTCTGGGCAAC	GTCACTGTCTTGGTTGTTGATG	AF286469
SCD-1	TGGGTTGGCTGCTTGTG	GTGTGGGCAGGATGAAG	NM 139192
FAS	TGCTCCCAGCTGCAGGC	GCCCGGTAGCTCTGGGTGTA	NM 017332
G3PDH	GCTTCGGTGACAACACCA	AGCTGCTCAATGGACTTTCC	NM 022215
LEPTIN	ATTTCACACACGCAGTCGGTATCCG	CCAGCAGATGGAGGAGGTC	NM 013076
PPAR*γ*	TCCTCCTGTTGACCCAGAGCAT	AGCTGATTCCGAAGTTGGTGG	???
LPL	GAGATTTCTCTGTATGGCACA	CTGCAGATGAGAAACTTTCTC	NM 012598
ADIPONECTIN	AATCCTGCCCAGTCATGAAG	CATCTCCTGGGTCACCCTTA	NM 144744

**Table 3:** Effect of dietary ALA content on content of n-6 and n-3 PUFA in red blood cell phospholipids.

	0.095%en ALA	0.19%en ALA	0.38%en ALA	0.76%en ALA	1.00%en ALA	2.05%en ALA	4.1%en ALA	6.3%en ALA
ALA	0.02 ± 0.001^a^	0.03 ± 0.001^a^	0.04 ± 0.004^a^	0.10 ± 0.004^b^	0.11 ± 0.003^b^	0.24 ± 0.002^c^	0.47 ± 0.01^d^	0.89 ± 0.02^e^
LA	4.86 ± 0.07^a^	5.09 ± 0.07^a^	5.21 ± 0.08^ab^	5.63 ± 0.13^bc^	5.96 ± 0.04^cd^	6.12 ± 0.07^d^	6.87 ± 0.13^e^	7.67 ± 0.16^f^
AA	27.9 ± 0.2^f^	27.9 ± 0.3^ef^	27.9 ± 0. 2^f^	26.3 ± 0.4^cd^	26.1 ± 0.79^bc^	24.6 ± 0.41^b^	21.7 ± 0.17^a^	20.8 ± 0.18^a^
EPA	0.88 ± 0.01^a^	0.13 ± 0.004^a^	0.24 ± 0.01^a^	0.54 ± 0.01^a^	0.66 ± 0.01^b^	1.40 ± 0.03^c^	2.63 ± 0.05^d^	3.46 ± 0.12^e^
DPA	0.63 ± 0.01^a^	0.76 ± 0.02^b^	0.98 ± 0.03^c^	1.30 ± 0.04^e^	1.57 ± 0.01^d^	2.22 ± 0.02^f^	2.40 ± 0.05^g^	3.06 ± 0.10^h^
DHA	1.63 ± 0.03^ab^	1.86 ± 0.05^c^	2.05 ± 0.04^d^	2.04 ± 0.10^d^	2.12 ± 0.05^d^	2.05 ± 0.03^d^	1.68 ± 0.04^b^	1.43 ± 0.06^a^
Total n6 PUFA	33.3 ± 0.18^c^	33.6 ± 0.26^c^	33.8 ± 0.07^c^	32.8 ± 0.77^bc^	32.6 ± 0.32^bc^	31.4 ± 0.40^b^	29.2 ± 0.25^a^	29.1 ± 0.23^a^
Total n3 PUFA	2.48 ± 0.04^a^	2.89 ± 0.06^a^	3.45 ± 0.04^b^	4.11 ± 0.13^c^	4.59 ± 0.04^c^	6.05 ± 0.063^d^	7.66 ± 0.07^e^	9.12 ± 0.28^f^
Total n3 LCPUFA	3.35 ± 0.04^a^	2.75 ± 0.06^a^	3.29 ± 0.04^b^	3.89 ± 0.13^c^	4.34 ± 0.04^c^	5.67 ± 0.06^c^	7.01 ± 0.08^e^	8.02 ± 0.26^f^
Total PUFA	38.5 ± 0.19	39.0 ± 0.20	39.6 ± 0.09	38.9 ± 0.81	39.1 ± 0.32	40.0 ± 0.38	39.3 ± 0.25	39.6 ± 0.51

All data are presented as a percentage of total lipids in the sample. Different superscripts denote values which are significantly different as determined by a Duncan's post hoc analysis (*P* < .05).

**Table 4:** Effect of dietary ALA content on the expression of SREPB1c, SCD-1, FAS, G3PDH, and leptin mRNA in omental and retroperitoneal adipose tissue.

	0.095%en ALA	0.19%en ALA	0.38%en ALA	0.76%en ALA	1.00% en ALA	2.05%en ALA	4.1%en ALA	6.3% en ALA
Omental Adipose Depot								

SREBP1c mRNA(×1000)	0.88 ± 0.09^cd^	0.74 ± 0.03^bc^	1.0 ± 0.2^d^	0.95 ± 0.02^d^	0.75 ± 0.10^cd^	0.60 ± 0.09^abc^	0.40 ± 0.03^ab^	0.34 ± 0.02^a^
SCD-1 mRNA	0.72 ± 0.06^ab^	0.43 ± 0.13^a^	1.22 ± 0.15^ab^	1.42 ± 0.23^ab^	0.67 ± 0.23^bc^	0.80 ± 0.19^c^	0.31 ± 0.11^a^	0.43 ± 015^a^
FAS mRNA	0.11 ± 0.02^bc^	0.06 ± 0.02^ab^	0.17 ± 0.03^c^	0.17 ± 0.008^c^	0.04 ± 0.02^ab^	0.05 ± 0.02^ab^	0.03 ±0.009^a^	0.06 ± 0.02^ab^
G3PDH mRNA	0.06 ± 0.003^a^	0.05 ± 0.02^a^	0.14 ± 0.02^b^	0.25 ± 0.01^c^	0.10 ± 0.02^ab^	0.10 ± 0.03^ab^	0.04 ± 0.004^a^	0.05 ± 0.02^a^
Leptin mRNA	0.01 ± 0.002	0.01 ± 0.004	0.02 ± 0.006	0.03 ± 0.005	0.02 ± 0.006	0.02 ± 0.006	0.01 ± 0.005	0.009 ± 0.002
PPAR*γ* mRNA	0.029 ± 0.003	0.021 ± 0.003	0.020 ± 0.004	0.030 ± 0.004	0.017 ± 0.009	0.018 ± 0.003	0.018 ± 0.005	0.016 ± 0.007
LPL mRNA	1.30 ± 0.26	1.30 ± 0.23	1.97 ± 0.44	1.73 ± 0.16	2.09 ± 0.62	1.47 ± 0.37	0.97 ± 0.36	0.89 ± 0.08
Adiponectin mRNA	0.74 ± 0.09	0.89 ± 0.15	1.57 ± 0.03	1.62 ± 0.08	3.20 ± 0.79	1.22 ± 0.34	1.56 ± 0.57	1.13 ± 0.44

Retroperitonl Adipose Depot								

SREBP1c mRNA (×10^4^)	0.25 ± 0.04^bc^	0.27 ± 0.05^bc^	0.39 ± 0.09^ c^	0.30 ± 0.08^bc^	ND	0.21 ± 0.02^ab^	0.16±0.06^ab^	0.11 ± 0.02^a^
SCD-1 mRNA	1.69 ± 0.41	1.24 ± 0.03	1.45 ± 0.34	1.60 ± 0.20	ND	1.92 ± 0.19	1.01 ± 0.24	0.92 ± 0.11
FAS mRNA	0.11 ± 0.01^b^	0.08±0.009^b^	0.09 ± 0.01^b^	0.11 ± 0.02^b^	ND	0.11 ±0.01^b^	0.03 ± 0.004^a^	0.04 ± 0.009^a^
G3PDH mRNA	0.30 ± 0.03^ab^	0.33 ± 0.03^ab^	0.29 ± 0.03^ab^	0.40 ±0.07^b^	ND	0.41 ± 0.03^b^	0.23 ± 0.03^a^	0.23 ± 0.02^a^
Leptin mRNA	0.04 ± 0.009	0.05 ± 0.008	0.04 ± 0.009	0.06 ± 0.01	ND	0.05 ± 0.006	0.04 ± 0.005	0.03 ± 0.008

Expression of all genes was normalised to the expression of *β*-actin. Different superscripts denote values which are significantly different as determined by a Duncan's post hoc analysis (*P* < .05); ND = no data.

**Table 5:** Summary of the relationships between expression of SREPB1c mRNA in omental and perineal fat and AA, total n-6 PUFA, EPA, DPA, DHA, and total n-3 LCPUFA consent of erythrocyte phospholipids as a % of total fatty acids.

	SREBP1c omental fat	SREBP1c retroperitoneal fat
DHA	ns	ns
EPA	*r* ^2^ = 0.62, *P* < .0001	*r* ^2^ = 0.22, *P* < .005
DPA	*r* ^2^ = 0.59, *P* < .0001	*r* ^2^ = 0.19, *P* < .005
Total omega-3 LCPUFA	*r* ^2^ = 0.59, *P* < .0001	*r* ^2^ = 0.19, *P* < .005
AA	*r* ^2^ = 0.66, *P* < .0001	*r* ^2^ = 0.21, *P* < .005
Total n-6	*r* ^2^ = 0.71, *P* < .0001	*r* ^2^ = 0.20, *P* < .005

All relationships were determined by simple linear regression. Relationships between SREBP-1c mRNA expression and DPA, EPA and total omega-3 LCPUFA were all negative, whilst relationships with AA and total n-6 PUFA and SREBP-1c mRNA were positive.
